# Walking Recovery after a Hip Fracture: A Prospective Follow-Up Study among Community-Dwelling over 60-Year Old Men and Women

**DOI:** 10.1155/2014/289549

**Published:** 2014-01-06

**Authors:** Anu Salpakoski, Timo Törmäkangas, Johanna Edgren, Sanna Sihvonen, Mika Pekkonen, Ari Heinonen, Maija Pesola, Mauri Kallinen, Taina Rantanen, Sarianna Sipilä

**Affiliations:** ^1^Department of Health Sciences, Gerontology Research Center, University of Jyväskylä, P.O. Box 35, 40014 Jyväskylä, Finland; ^2^School of Health and Social Studies, University of Applied Sciences, Rajakatu 35, 40200 Jyväskylä, Finland; ^3^Peurunka-Medical Rehabilitation and Physical Exercise Center, Peurungantie 85, 41340 Laukaa, Finland; ^4^Department of Health Sciences, University of Jyväskylä, P.O. Box 35, 40014 Jyväskylä, Finland; ^5^Department of Orthopedics and Traumatology, Central Finland Central Hospital, Keskussairaalantie 19, 40620 Jyväskylä, Finland; ^6^Department of Medical Rehabilitation, Oulu University Hospital, P.O. Box 21, 90029 Oulu, Finland

## Abstract

*Purpose*. Recovery of walking outdoors after hip fracture is important for equal participation in the community. The causes of poor recovery are not fully understood. This study investigates recovery of walking outdoors and associated determinants after hip fracture. *Methods*. A prospective follow-up study, among clinical sample of 81 community-dwelling hip fracture patients over 60 years. Perceived difficulty in walking outdoors and 500 meters was assessed before fracture, at discharge to home (3.2 ± 2.2 weeks after surgery), and on average 6.0 ± 3.3 weeks after discharge. Potential determinants for walking recovery were assessed. Linear latent trajectory model was used to analyse changes during follow-up. Association between walking trajectories and potential determinants was analysed with a logistic regression model. *Results*. Two trajectories, No-to-minor-difficulty and Catastrophic, were found. Thirty-eight percent of the participants ended up in the Catastrophic trajectory for walking outdoors and 67% for 500 meters. Multivariate logistic regression analysis revealed that use of walking aid and indoor falls before fracture and prolonged pain were independently associated with catastrophic decline in both primary outcomes: difficulty in walking outdoors and 500 meters. *Conclusions*. A large proportion of community-dwelling older people recovering from hip fracture experienced catastrophic decline in outdoor walking. Acknowledging recovery prognoses at early stage enables individualized rehabilitation.

## 1. Introduction

Poor recovery after hip fracture causes considerable suffering for the patients and imposes a financial burden on the social and health care sector. Recovery of walking ability after hip fracture is a necessity for reestablishing patients into their normal environment. The causes of poor recovery are not fully understood. A few studies have investigated walking recovery after the hip fracture [1–3]. Taylor et al. [3] reported that, during inpatient rehabilitation, on average four weeks after hip fracture, patients experienced improvement in walking ability and physical factors, independent of pain or balance. However, on average three months after discharge, they reported pain, poor balance, and fear of falling, as well as reduced outdoor mobility, walking ability, and participation in community activities.

Moving outdoors is essential for the independence of community-dwelling older people. Difficulties in walking outdoors raise inequality issues as sufficient walking ability is needed for the access to public services and participation in the community. Therefore, it is important to eliminate all the barriers for outdoor walking [4]. The ability to walk even a short distance outdoors can be meaningful for successful and independent living at home. Moreover, among persons with walking limitations, outdoor activities might help to maintain physical functioning [5].

At discharge from the hospital, community-dwelling hip fracture patients usually receive a written home exercise program. However, neither compliance with the program nor mobility recovery after the fracture is systematically followed up [6]. The first weeks after hip fracture have been found to be critical for the recovery. But the lack of supported discharge, long-term follow-up, and planned individualized long-term rehabilitation is acknowledged [7].

The aim of this study was to investigate recovery of walking outdoors within the first ten weeks after hip fracture among over 60-year-old community-dwelling men and women. In addition, we explored the determinants associated with the different walking trajectories after hip fracture.

## 2. Materials and Methods

### 2.1. Participants

This study utilizes baseline data of a randomized controlled trial (ISRCTN53680197). This is a prospective follow-up design covering on average the first ten post hip fracture weeks and retrospective data on prefracture walking difficulties. A detailed description of the study design and recruitment of the participants has been published earlier [6]. Briefly, the medical records of hip fracture patients were reviewed between 1.3.2008 and 31.12.2010. All patients fulfilling the inclusion criteria (>60 years, ambulatory, community-dwelling, operated for femoral neck or trochanteric fracture, and living in the ten municipalities in Central Finland) were informed about the study during the inpatient period after surgery (*n* = 296). Of those, 161 were interested in the study and were further visited by one of the researchers. Finally, 136 persons were recruited. Patients suffering from severe memory problems (MMSE < 18), alcoholism, severe cardiovascular, pulmonary, or a progressive disease, or severe depression (BDI-II > 29) were excluded. In total, 81 persons participated in the study.

### 2.2. Ethical Approval

The study was conducted in accordance with the ethical principles stated in the Declaration of Helsinki. The ethical committee of the Central Finland Health Care District approved the study (K-Sshp Dnro56/2007). All participants gave their written informed consent prior to participating in the study.

### 2.3. Measurements

The primary outcomes of the study, self-reported difficulties in walking outdoors and 500 meters, were assessed at three different time points: (1) before fracture elicited at the hospital on average 10 ± 5 days after fracture, (2) at discharge from the hospital or health care centre, and (3) 6 ± 3 weeks after discharge to home. The questions were formulated as “Do you have difficulty in walking outdoors/500 meters?” with the following response options: (1) able to manage without difficulty, (2) able to manage with some difficulty, (3) able to manage with a great deal of difficulty, (4) able to manage only with the help of another person, and (5) unable to manage even with help. For the trajectory analyses, options 4 and 5 were combined due to the low response frequencies in the latter category.

#### 2.3.1. Prefracture Information

Demographics and chronic diseases present for at least three months were collected from the medical records of hospital and health care centres. The comorbidity was calculated as the number of chronic diseases. In addition, diagnoses of the osteoarthritis, osteoporosis, and diabetes were reported. Use of walking aids outdoors and falls indoors and outdoors during the previous year were collected with a questionnaire during the inpatient period. Falls were dichotomised as “no falls” and “one or more falls”.

#### 2.3.2. Hospital Information

Hip fracture diagnosis, type of surgery, and the lowest haemoglobin value after surgery were collected from the medical records. Type of surgery was categorised as fixation (internal fixation of femoral neck and extra-/intramedullary fixation of trochanteric fracture), hemiarthroplasty and total hip replacement. Time from fracture to surgery, duration of inpatient period and time from discharge to laboratory assessments are reported.

#### 2.3.3. Information at Discharge

As early mobilization after hip fracture is associated with walking recovery [8], we assessed perceived difficulties in walking at the hospital ward. The question was formulated as follows: “How do you manage moving around in the ward?” The response options were (1) able to manage without difficulty, (2) able to manage with some difficulty, (3) able to manage with great deal of difficulty, (4) able to manage only with the help of another person, and (5) unable to manage even with help. These were dichotomised into “no difficulty” (1) and “difficulty” (2)–(5). Moving-related pain on the fractured side was assessed with the question “Do you have offending pain in the low back/hip/knee on the fractured side which impairs your moving?” Pain was considered moving related if it affected moving in at least one of the sites at discharge. The presence of chronic diseases and use of prescription medication, including painkillers, were confirmed according to a prestructured questionnaire, prescriptions, and medical records.

#### 2.3.4. Information 6 Weeks after Discharge

The physical performance measurements were performed in the research laboratory. Contraindications for safe participation were evaluated by a physician [9].

Functional balance was measured using the Berg Balance Scale [10], which evaluates an individual's ability to perform different tasks related to the skills of sitting down, standing up, reaching, turning around, looking over one's shoulder, and one-foot standing. The ability to perform each of the 14 tasks is rated from 0 (incapable) to 4 (safe and independent). The maximum score is 56, and higher scores indicate better functional balance.

Maximal isometric knee extension force was measured in the fractured and nonfractured side using an adjustable dynamometer chair (Good Strength, Metitur LTD, Palokka, Finland). In the statistical analysis knee extensor force was adjusted with the body weight. The ankle was attached to a strain-gauge with the knee angle fixed at 60 degrees from full extension. The leg was extended as forcefully as possible and participants were encouraged to make a maximal effort during the measurement. The measurement was repeated at least three times until no further improvement occurred. The best performance was used in the analysis. Maximal isometric handgrip force of the dominant hand was measured using an adjustable dynamometer chair (Good Strength, Metitur LTD, Palokka, Finland). The dynamometer was fixed to the arm of the chair with the elbow angle of 90 degrees. The handle was squeezed as hard as possible and the measurement was repeated at least three times until no further improvement occurred. The best performance was used in the analysis.

The amount of pain in the low back, hip, and knee region on both sides of the body during the last week was assessed with the Visual Analog Scale (VAS) [11]. A 100 mm line without numbers was used. A summary index was calculated from all six VAS variables. Body weight was measured in kilograms and height in centimetres.

### 2.4. Statistical Analyses

For trajectory analysis the growth mixture model approach was used, which permits the identification of population subgroups following similar recovery trajectories and separates them from other subgroups. The key property of the approach is that the subgroups are not known in advance, but their presence is inferred from the data. A mixture model includes two (or more) latent groups, whose trajectory parameters are estimated simultaneously together with each individual's probabilities of belonging into these groups. The membership probabilities can be used to describe the clarity of differences between the two groups via the summary statistic entropy, which ranges between zero and one, where a value close to one indicates an unambiguous grouping [12]. Growth mixture models have recently been used in the analysis of trajectories of various variable types [13–17] but less for ordered-category outcomes. Within the latent subgroups a growth model was fitted to the data while allowing the model growth parameters to vary over the latent groups. Within latent class *g*  (*g* = 1,2), the (scaled) threshold structure of the response variables within time-point *i*  (*i* = 1,…, 3) and response category *j*  (*j* = 1,…, 3) was modeled according to

(1) $$\begin{array}{c} y_{(G)ij}=\mu_{G}+0.1c_{i}\beta_{1}+{\left(0.1{\text{c}}_{i}\right)}^{2}\beta_{2}+c_{j}b_{ij}\tau_{i}, \end{array}$$

where *μ* is the grand mean and *β*_1_ and *β*_2_ are the linear and quadratic growth coefficients, respectively. The coefficient *τ* describes the separation of the categories within the time points, and we permitted the separation to vary according to time by estimating threshold-specific factor *b*. At the first time point we fixed *b* to one for the first response category within the latent groups to establish this as the reference category. The design vector *c* = (−1,0, 1) represents the time point-specific contrast. This model structure enables the structure of the trajectories to be separated into growth parameters, both linear and quadratic, as well as other types of differences in the response probabilities, which we will refer to as nonlinear. The maximum likelihood estimator used in the analyses permitted retaining the participant in analysis, if there was a nonmissing measurement from at least one time point.

Due to the small sample size we employed a three-stage strategy in the analysis of the walking recovery data. First, a growth mixture model was performed. Secondly, we compared the trajectory group differences for each determinant individually using the nonparametric median test for continuous variables and the chi-square test for discrete variables. Nonparametric tests were used because of nonnormality of the continuous measures. Thirdly, logistic regression modeling was performed to assess determinants, which were individually associated with walking trajectories. Determinants which differed significantly between the trajectories were included into the model, except for the frequency of osteoarthritis in walking 500 meters. We observed that logistic regression with osteoarthritis as a predictor led to quasi-complete separation, where the maximum likelihood parameter estimate does not exist [18]. The problem is due to the frequencies of diagnoses of osteoarthritis in the lower extremity: 19% in the Catastrophic trajectory group and none in the No-to-minor trajectory group. In Model I each determinant was a single regression of the probability of being included in the Catastrophic trajectory group. In Model II the Catastrophic trajectory group probability was predicted by all statistically significant variables at the study time points (before fracture, discharge, six weeks after discharge). All models were adjusted for age and gender. In addition, the C-statistic was calculated and used as the discrimination index in the fully adjusted logistic regression models.

The trajectory models were fitted with the Mplus-program (version 6; Muthén & Muthén 2011). Descriptive statistics and group comparisons were carried out using the R-environment (version 2.12.2; R Development Core Team 2011). The chi-square tests of independence were performed using the R-package gmodels (version 2.15.1), C-statistics with R-package rms (version 3.6.3), and logistic regression with IBM SPSS Statistics for Windows (version 19.0; Armonk, NY:IBM Corp.).

## 3. Results

Sixty-three (78%) of 81 participants were women and the average age was 80.0 ± 7.1 years. Fifty-two participants (64%) had a femoral neck fracture and 29 a trochanteric fracture. Time from fracture to surgery was on average 3 ± 4 days, duration of inpatient period 3.2 ± 2.2 weeks, and time from discharge to the laboratory assessments 6.0 ± 3.3 weeks.

Walking outdoors and walking 500 meters were assessed from 79 (98%), 73 (90%), and 81 (100%) participants in the three measurement waves. In walking outdoors (*P* < 0.001) and walking 500 meters (*P* < 0.001), the parametric bootstrapped likelihood ratio test [19] indicated that two groups were more likely than one to account for the difficulty trajectories. We called these trajectories the No-to-minor-difficulty and Catastrophic trajectories. It is noteworthy that mobility difficulties increased in both groups and, among majority of the participants, recovery to the prefracture state was not observed.

### 3.1. Walking Outdoors

**Figure 1:**
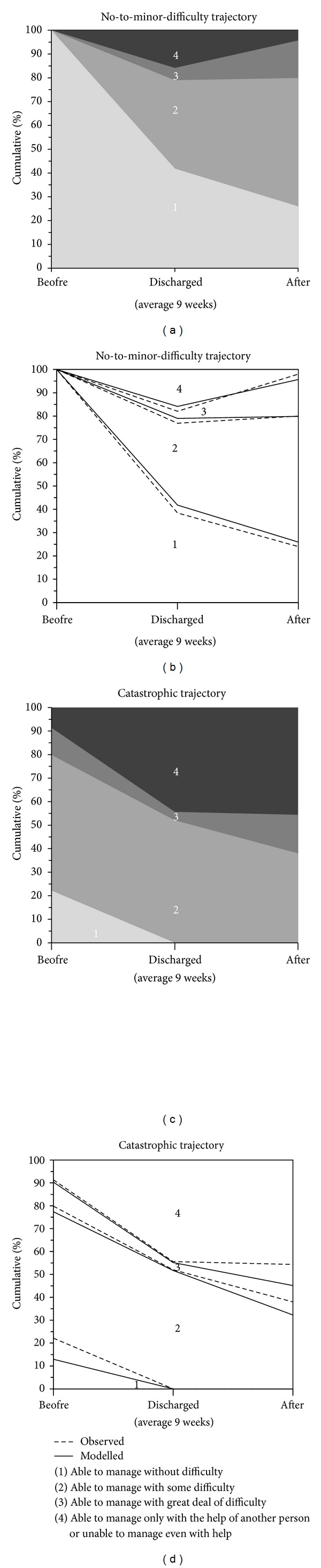
Trajectories for walking outdoors. The figures show the cumulative percentage of individuals within a response category at the three time points. Upper ((a)-(b)) No-to-minor-difficulty trajectory and lower ((c)-(d)) Catastrophic trajectory. Panels (b) and (d) show amount of participants actually observed and modelled.

Figure 1 shows the trajectories for difficulties in walking outdoors (entropy: 0.73), along with the cumulative percentage of individuals within a response category at each of the three time points. We found that in the No-to-minor-difficulty trajectory (*n* = 50) there was a curvilinear development (linear *P* = 0.003; quadratic *P* = 0.004), which is seen as initial drop in and levelling-off of the cumulative percentages. This trajectory included participants with no prefracture mobility difficulties. At discharge, 42% of them and at six weeks thereafter 26% reported no difficulties in walking outdoors. In the Catastrophic trajectory (*n* = 31), we observed linear worsening (*P* = 0.001) over time. Twenty-two percent of the participants in the Catastrophic trajectory reported no difficulties before fracture. Mobility difficulties increased at discharge and continued to increase six weeks thereafter. Nearly half of the participants in the Catastrophic trajectory needed help of another person in walking outdoors at discharge (44%) and at 6 weeks after discharge (46%).

### 3.2. Walking 500 Meters

**Figure 2:**
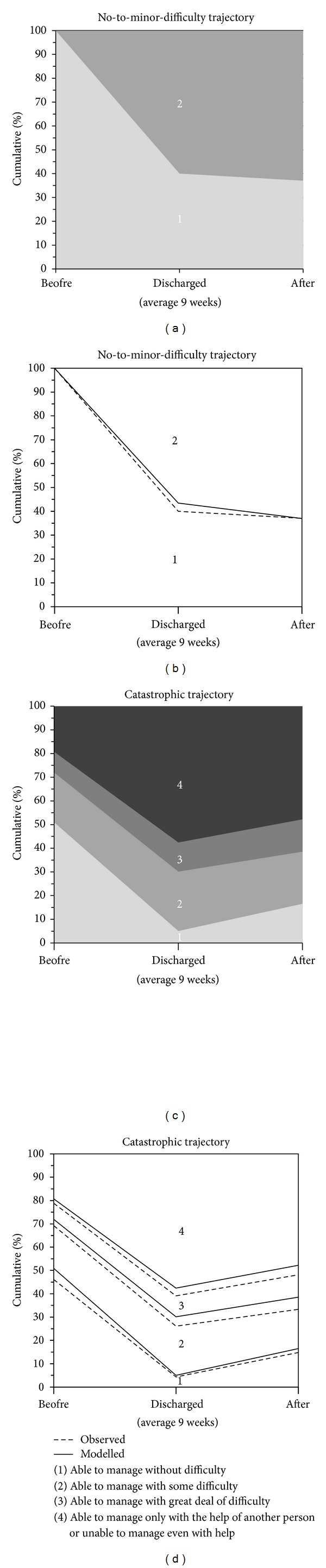
Trajectories for difficulties in walking 500 meters. The figures show the cumulative percentage of individuals within a response category at the three time points. Upper ((a)-(b)) No-to-minor-difficulty trajectory and lower ((c)-(d)) Catastrophic trajectory. Panels (b) and (d) show amount of participants actually observed and modelled.

Figure 2 shows the cumulative percentages of the trajectory model for difficulties in walking 500 meters (entropy: 0.78). Overall, the No-to-minor-difficulty trajectory (*n* = 27) had low reported levels of difficulties, and the trajectory structure was explained by linear decline (*P* = 0.007). None of the participants in the No-to-minor-difficulty trajectory had prefracture walking difficulties. At discharge, 57% and six weeks thereafter 63% of the participants reported some difficulties. In the Catastrophic trajectory group we observed a curvilinear development (linear *P* < 0.001; quadratic *P* = 0.004). In the Catastrophic trajectory, 19% were unable to walk 500 meters without the help of another person before fracture. Perceived difficulties increased dramatically after the fracture, as at discharge 58% and at 6 weeks after discharge 48% needed the help of another person.

### 3.3. Differences in the Trajectory Groups

Underlying determinants associated with the trajectories in walking outdoors (Table 1) were higher frequencies in the use of walking aids and indoor falls during the previous year, longer hospitalisation period, and more offending pain at discharge in the Catastrophic compared to the No-to-minor-difficulty group. At six weeks after discharge, the participants in the Catastrophic trajectory reported significantly more pain in the lower body than those in the No-to-minor-difficulty trajectory. Compared to the participants in the Catastrophic trajectory, functional balance was better and the knee extensor force of the non-fractured side greater in the No-to-minor-difficulty trajectory, reflecting worse overall physical function in the Catastrophic trajectory weeks after returning to live in the community.

Participants using walking aids before fracture had nearly eight times the risk, those who fell indoors prior to the fracture nearly four times the risk, and those suffering from offending pain at discharge nearly six times the risk for Catastrophic trajectory, compared to those who had no walking aids or who had not fallen indoors before fracture or who did not suffer from pain at discharge. In addition, longer inpatient period and pain six weeks after the discharge were significantly associated with ending up in Catastrophic trajectory.

In walking 500 meters (Table 2), the participants in the Catastrophic trajectory were older than those in the No-to-minor-difficulty trajectory. The prefracture use of walking aids, indoor falls, and lower extremity osteoarthritis were more common among the Catastrophic trajectory group. Time from surgery to discharge was longer among the participants in the Catastrophic trajectory compared to the No-to-minor-difficulty trajectory group. Six weeks after discharge, the participants in Catastrophic trajectory reported more pain in the lower body and had poorer functional balance than those in the No-to-minor-difficulty trajectory. Logistic regression analyses revealed that the risk for Catastrophic trajectory was nearly six times greater among those who used walking aids before fracture and nearly five times greater among those who fell indoors before fracture compared to those with no walking aids or indoor fall history pre-fracture. Additionally, longer inpatient period and pain six weeks after the discharge were associated with risk for ending up in Catastrophic trajectory.

## 4. Discussion

At six weeks after discharge to home from the inpatient period, the participants of this study reported severe difficulties in walking outdoors. Two trajectories for the recovery of walking outdoors and 500 meters were found: No-to-minor-difficulty trajectory and Catastrophic trajectory. The No-to-minor-difficulty trajectory included participants with no pre-fracture walking difficulties, followed by some difficulties after fracture. Majority of the participants in the Catastrophic trajectory had prefracture outdoor walking difficulties followed by a steep decline after fracture. The underlying determinants associated with the Catastrophic walking trajectories were use of walking aids and indoor falls before the fracture, length of the inpatient period, offending pain on the fractured limb at discharge, and prolonged lower body pain.

Our results are in line with the previous studies showing that walking recovery after the hip fracture is challenging [2, 20, 21]. We observed that, on average, six weeks after returning home, the majority of the participants reported difficulty in walking outdoors and 500 meters and nearly half of the participants in the Catastrophic trajectory needed the help of another person. A large proportion of older community-dwelling people recovering from hip fracture, are home bound and immobile [22]. During the recovery process, the first steps outside the home may be taken at the porch, patio, backyard, or driveway. Walking for a longer distance, such as 500 meters or one block is, however, necessary for participation in common societal activities such as use of services, shopping, or leisure time activities. According to our results, approximately two-thirds of the participants did not end up in catastrophic decline in outdoor walking ability and were thus able to return to outdoor activities near home with minor difficulties. However, two-thirds of the participants experienced a catastrophic decline in the ability to walk 500 meters suggesting severe difficulties in returning to community activities after hip fracture. These results suggest that current rehabilitation strategies do not sufficiently take into account the prerequisites for safe walking and the ability to return to community activities. In addition, we know that difficulty or the inability to walk outdoors increases the risk for dependence and institutionalization [23]. Hip fractures cause considerable health care costs during the first postfracture year [24–26]. The cost burden can double or even triple if a home-dwelling person is admitted to permanent institutional care because of a fracture [26, 27].

Characterization of the determinants associated with poor outdoor walking recovery after hip fracture is important. Previous knowledge on walking recovery in general is fragmentary and the factors associated with poor recovery of walking outdoors have been even less investigated. Previous studies suggest that prefracture use of walking aids [1, 28], high age [29, 30], handgrip strength at admission to hospital [31], and longer inpatient period [32] predict recovery of the physical function after hip fracture. In the present sample, some of the determinants associated with increased difficulty in walking outdoors were present before the fracture. The individuals in the Catastrophic trajectory were more often older, had more indoor falls prior to the fracture, and were more likely to have osteoarthritis in the lower extremity and to use walking aids than those in the No-to-minor-difficulty trajectory. The results revealed that the risks for ending up in the Catastrophic trajectory were 6 to 8 times higher, if the participant used walking aid outdoors and about four times higher, if the participant had fell indoors in the previous year before the hip fracture. A recent study by Gill et al. [33] also found out that the change in functional status before an injurious fall was associated with functional recovery trajectories a year after the fall. Contrary to our results previous studies have suggested that factors related to the clinical condition of the patient, gender, or treatment of the fracture may predict poor recovery after a hip fracture [1, 29, 30, 32, 34–38]. In this study, only the duration of inpatient care was significantly longer among the Catastrophic than No-to-minor-difficulty group. A longer inpatient period may reflect poor health status or complications during the acute treatment of the fracture. However, we did not find differences between the groups in either the burden of chronic diseases or postoperative haemoglobin concentration.

Earlier studies have shown that older people suffer from persistent muscle weakness [39], balance impairment [40], and pain [41] after hip fracture. It is well known that these factors are associated with walking limitation and disability among older populations. The participants in the Catastrophic group had poorer postural balance, knee extension force, and more pain than those in the No-to-minor-difficulty group. Pain in the low back, hip, and knee regions was particularly high in the Catastrophic group. They also experienced offending pain in the lower body and the risk for ending up in the Catastrophic trajectory in walking outdoors was almost six times greater, if the participant experienced offending pain on the fractured limb at discharge. Severe pain often leads to physical inactivity [41], restriction of painful movements, and fear of pain, all of which may induce even more pain and further avoidance of activity. The coexistence of walking disability, pain, poor balance, and muscle weakness predicts loss of independence in the near future [2, 42, 43].

At discharge, participants of this study received a written home exercise program, which is part of the standard care. However, recovery of walking or compliance to the program was not systematically followed up [6]. Rehabilitation after hip fracture is important and should be extended to home after discharge. Currently, there are no accurate rehabilitation guidelines for walking recovery after hip fracture.

We investigated perceived difficulties in walking outdoors among 81 consecutive community-dwelling over 60-year-old hip fracture patients. Those who were too frail to travel to the laboratory assessments and who had severe memory problems or depression were excluded from the study. Therefore, our sample is not thoroughly representative among all hip fracture patients. Participants were followed up during the critical time for recovery, on average two months after fracture and six weeks after discharge. Perceived difficulty in walking outdoors before the fracture was assessed at the hospital. We believe, however, that our participants were able to recall their before fracture condition as this was assessed within the first ten days after fracture.

In clinical populations, the recruitment of participants is challenging, especially in studies where frequent travelling is required or where long-term interventions are incorporated into the study design. In the present study, the number of participants was based on sample size calculations of the RCT design with mobility recovery as the main outcome [6]. Therefore, it might be that the present secondary analysis is underpowered to detect all predictors of walking recovery. Owing to sample size, we modelled two trajectories for early outdoor walking recovery, which fitted the data well. With a larger sample size more than two trajectories might have been found. However, we believe that with this data we have identified the most important paths characterising early walking limitation after hip fracture and the key determinants defining outdoor walking recovery. Our main outcomes were perceived difficulty in walking outdoors and walking 500 meters, which reflects the individual's own understanding of her or his ability to cope with her or his own environment. This perspective, which has been more rarely investigated [3], is important, when defining the independence and capacity of the older people recovering from a hip fracture to participate in the community.

## 5. Conclusions

The determinants of severe disability in outdoor walking need to be acknowledged in clinical practice in order to design effective and individualized rehabilitation strategies and to prevent disability and institutionalization. These factors are easy to assess in clinical practice and some are modifiable by targeted rehabilitation. Older hip fracture patients with poor prognosis for outdoor walking recovery need special attention, systematic physical rehabilitation, and pain management to promote their participation and independent living in the community.

## Figures and Tables

**Table 1:** Characteristics of participants by walking trajectories in  walking  outdoors  (*n*, mean ± SD, median *P* value/*n* (%), *χ* ^2^ *P* value) and binary logistic regression model for statistically significant variables as a predictor for catastrophic trajectory.

	Trajectory					Binary logistic regression	
	No-to-minor-difficulty *n* = 50		Catastrophic *n* = 31		*P* value	Model I^e^	Model II^f^
	*n*		*n*			OR (95% CI)	OR (95% CI)
***Demographic and prefracture information***							
Age	50	78.6 ± 7.2	31	82.2 ± 6.5	*0.362 *		
Women (%)	50	42 (84)	31	21 (68)	*0.087 *		
Body height (cm)	50	160.9 ± 8.3	30	160.1 ± 10.0	*0.489 *		
Body weight (kg)	50	65.9 ± 11.6	31	65.6 ± 11.5	*1 *		
Number of chronic diseases	50	3.0 ± 1.7	31	3.8 ± 1.7	*0.343 *		
Osteoarthritis	50	5 (10)	31	5 (16)	*0.415 *		
Osteoporosis	50	4 (8)	31	6 (19)	*0.131 *		
Diabetes	50	4 (8)	31	5 (16)	*0.258 *		
Walking aid outdoors before fracture (%)	46	**16 (35)**	30	**23 (77)**	<***0.001***	**6.55 (2.05**–**20.96)**	**7.91 (2.20**–**28.51)**
Falling indoors, year before the fracture	49	**8 (16)**	31	**12 (39)**	***0.024***	**4.19 (1.32**–**13.32)**	**3.74 (1.04**–**13.51)**
Falling outdoors, year before the fracture	49	16 (33)	31	12 (39)	*0.580 *		
C-statistic of the model							0.82
***Hospital information***							
Collum fracture, S72.0 (%)	50	31 (62)	31	21 (68)	*0.600 *		
Type of surgery (%)	50		31		*0.338 *		
Fixation		24 (48)		14 (45)			
Hemiarthroplasty		18 (36)		15 (48)			
Total hip replacement		8 (16)		2 (6)			
Lowest haemoglobin after surgery (g/L)	48	98.8 ± 12.6	29	96.0 ± 14.7	*0.356 *		
Time from fracture to surgery (days)	50	3.1 ± 5.3	31	2.1 ± 1.9	*0.821 *		
***Information at discharge***							
Difficulties in walking at the ward (%)	44	3 (7)	30	2 (7)	*0.980 *		
Offending pain at discharge (%)	43	**12 (28)**	28	**17 (61)**	***0.006***	**5.97 (1.89**–**18.89)**	**5.66 (1.70**–**18.82)**
Duration of inpatient period (days)	50	18 ± 12	31	29 ± 18	***0.006***	**1.05 (1.01**–**1.09)**	**1.04 (1.00**–**1.09)**
C-statistic of the model							0.79
***Information 6 weeks after the discharge***							
Prescribed pain medication	50	27 (54)	31	23 (74)	*0.069 *		
Lower body pain (VAS)^d^	50	69.9 ± 77.9	30	169.8 ± 148.2	***0.002***	**1.09 (1.04**–**1.15)^g^**	**1.10 (1.03**–**1.17)^g^**
Functional balance (score)^c^	48	45.3 ± 6.2	30	36.9 ± 11.5	***0.002***	**0.89 (0.82**–**0.96)**	0.93 (0.85–1.02)
Knee extension force, nonfractured side (N)	49	249.6 ± 91.2	29	208.2 ± 77.8	***0.029^a^***	**0.92 (0.85**–**0.98)^g^**	0.93 (0.84–1.02)^g^
Knee extension force, fractured side (N)	46	187.5 ± 73.2	28	157.2 ± 67.9	*0.059^a^*		
Handgrip force (N)	49	205.7 ± 84.2	30	183.2 ± 79.2	*0.359 *		
Time in home-dwelling (days)	50	41.3 ± 13.3	31	43.3 ± 33.1	*0.067 *		
C-statistic of the model							0.86

^a^ *P* value is adjusted with the body weight using marginal means.

^b^ADL: activities of daily living.

^c^BBS: range 0–56.

^d^VAS: range 0–600.

^e^Model I: OR for prediction in logistic regression for statistically significant variables adjusted for age and gender.

^f^Model II: OR for prediction in logistic regression for all statistically significant variables from Model I in one time point, adjusted for age and gender.

^g^VAS and knee extension force were divided by 10 for the regression analysis.

Statistically significantly different values between the study groups are bolded and *P* values are in italic.

**Table 2:** Characteristics of participants by walking trajectories in  walking 500 meters  (*n*, mean ± SD, median  *P* value/*n* (%), *χ* ^2^ *P* value) and binary logistic regression model for statistically significant variables as a predictor for catastrophic trajectory.

	Trajectory					Binary logistic regression	
	No-to-minor-difficulty *n* = 27		Catastrophic *n* = 54		*P* value	Model I^e^	Model II^f^
	*n*		*n*			OR (95% CI)	OR (95% CI)
***Demographic and prefracture information***							
Age	27	77.9 ± 6.4	54	81.0 ± 7.3	***0.035***		
Women (%)	27	23 (85)	54	40 (74)	*0.257 *		
Body height (cm)	27	160.9 ± 8.1	53	160.4 ± 9.4	*1 *		
Body weight (kg)	27	64.9 ± 10.1	54	66.3 ± 12.3	*0.485 *		
Number of chronic diseases	27	3.1 ± 1.8	54	3.5 ± 1.7	*0.634 *		
Osteoarthritis	27	**0 (0)**	54	**10 (19)**	***0.017^h^***		
Osteoporosis	27	2 (7)	54	8 (15)	*0.339 *		
Diabetes	27	1 (4)	54	8 (15)	*0.134 *		
Walking aid outdoors before fracture (%)	25	**6 (24)**	51	**33 (65)**	<***0.001***	**5.77 (1.83**–**18.20)**	**5.49 (1.70**–**17.77)**
Falling indoors, year before the fracture	27	**3 (11)**	53	**17 (32)**	***0.041***	**4.49 (1.11**–**18.10)**	**4.64 (1.01**–**21.36)**
Falling outdoors, year before the fracture	27	8 (39)	53	20 (38)	*0.472 *		
C-statistic of the model							0.79
***Hospital information***							
Collum fracture, S72.0 (%)	27	20 (74)	54	32 (59)	*0.190 *		
Type of surgery (%)	27		54		*0.454 *		
Fixation		11 (41)		27 (50)			
Hemiarthroplasty		11 (41)		22 (41)			
Total hip replacement		5 (18)		5 (9)			
Lowest haemoglobin after surgery (g/L)	26	99.7 ± 14.7	51	96.7 ± 12.7	*1 *		
Time from fracture to surgery (days)	27	2.2 ± 1.9	54	3.0 ± 5.1	*0.347 *		
***Information at discharge***							
Difficulties in walking at the ward (%)	25	2 (8)	49	3 (6)	*0.761 *		
Offending pain at discharge (%)	25	7 (28)	46	22 (48)	*0.105 *		
Duration of inpatient period (days)	27	17 ± 13	54	26 ± 16	<***0.001***	**1.05 (1.00**–**1.10)**	**1.05 (1.00**–**1.10)**
***Information 6 weeks after the discharge^j^***							0.70
Prescribed pain medication	27	15 (56)	54	35 (65)	*0.419 *		
Lower body pain (VAS)^d^	27	54.9 ± 74.4	53	134.1 ± 129.0	***0.004***	**1.09 (1.02**–**1.16)^g^**	**1.07 (1.00**–**1.15)^g^**
Functional balance (score)^c^	26	46.8 ± 4.7	52	39.8 ± 10.4	***0.004***	**0.86 (0.77**–**0.96)**	0.90 (0.81–1.01)
Knee extension force, nonfractured side (N)	27	252.9 ± 85.4	51	224.3 ± 89.0	*0.056^a^*		
Knee extension force, fractured side (N)	26	191.0 ± 66.7	48	167.9 ± 74.6	*0.087^a^*		
Handgrip force (N)	20	210.0 ± 73.3	42	190.5 ± 86.9	*0.155 *		
Time in home-dwelling (days)	27	42.2 ± 12.7	54	41.9 ± 26.6	*0.235 *		
C-statistic of the model							0.79

^a^ *P* value is adjusted with the body weight using marginal means.

^b^ADL: activities of daily living.

^c^BBS: range 0–56.

^d^VAS: range 0–600.

^e^Model I: OR for prediction in logistic regression for statistically significant variables adjusted for age and gender.

^f^Model II: OR for prediction in logistic regression for all statistically significant variables from Model I in one time point, adjusted for age and gender.

^g^VAS and knee extension force were divided by 10 for the regression analysis.

^h^Logistic regression analysis impossible due to quasicomplete separation and because maximum likelihood parameter does not exist.

Statistically significantly different values between the study groups are bolded and *P* values are in italic.
